# The simple multivariable model for predicting liver fibrosis in Vietnamese male adults: a combination of Bayesian model averaging and stepwise method

**DOI:** 10.7717/peerj.20435

**Published:** 2026-01-14

**Authors:** Nghia Nhu Nguyen, Bao The Nguyen, Huyen Thi Ngoc Le, Hoang Nhat Dang, Hao Minh Pham, Duong Dai Ngo, Duy Khanh Tran Nguyen, Tam Thai Thanh Tran, Hung Thanh Kim, Tan Ngoc Huynh Mai

**Affiliations:** 1 Can Tho University of Medicine and Pharmacy, Can Tho, Vietnam; 2 121 Military Hospital, Can Tho, Vietnam

**Keywords:** Liver fibrosis, Bayesian model averaging, Stepwise, Predictive model

## Abstract

**Background:**

Liver fibrosis is a significant health burden in Vietnamese male adults, driven by high rates of hepatitis B and hepatitis C, excessive alcohol consumption, and genetic and environmental factors. Despite progress in diagnostic tools, there is a pressing need for cost-effective screening methods tailored to this high-risk group, particularly in resource-limited settings.

**Methods:**

This study enrolled 952 Vietnamese male adults over 40 years old undergoing FibroScan, excluding those with conditions affecting test accuracy. Data on demographics, clinical history, and anthropometrics were collected, and fibrosis stages were classified using the METAVIR system. Model development combined Bayesian model averaging and forward stepwise methods, with predictive performance validated *via* receiver operating characteristic (ROC) analysis and area under the curve (AUC) estimation in the R environment.

**Results:**

Among 952 male participants, the prevalence of liver fibrosis was 19.9%, with most cases classified as mild (F1). Multivariate analysis identified significant risk factors, including advanced age (odds ratio (OR) = 1.6; 95% confidence interval (CI) [1.02–2.51]), alcohol abuse (OR = 4.44; 95% CI [2.65–7.42]), hepatitis B (OR = 6.76; 95% CI [3.14–14.54], hepatitis C (OR = 33.04; 95% CI [5.26–207.42]), family history of cirrhosis (OR = 16.14; 95% CI [3.28–79.55]), and hepatic steatosis (OR = 4.02; 95% CI [2.57–6.28]). The predictive model demonstrated good discriminative performance with an AUC of 0.769 (95% CI [0.734–0.800]) and showed satisfactory calibration through bootstrap resampling, indicating close agreement between predicted and observed risks.

**Conclusion:**

The current prevalence of liver fibrosis among Vietnamese male adults was found to be 19.9%, and the developed risk prediction model effectively identifies high-risk individuals, enabling early diagnosis and targeted prevention, particularly in resource-limited settings. However, the lack of external validation and the sample restricted to Vietnamese male adults limit the generalizability of the model, which should be further evaluated in other populations.

## Introduction

Chronic liver disease is a significant global health burden, accounting for approximately 2 million deaths annually worldwide (Asrani et al., 2019; Roehlen, Crouchet & Baumert, 2020). Among the primary causes of chronic liver disease, viral hepatitis (particularly hepatitis B and C) and alcoholic hepatitis play critical roles (Lavanchy, 2009; Razavi-Shearer et al., 2018; Terrault et al., 2018; Younossi et al., 2016). Chronic liver diseases are often associated with the continuous destruction and regeneration of liver cells, leading to liver fibrosis and eventually progressing to liver cirrhosis (Berumen et al., 2021).

Fibrosis of organs is a hallmark of progressive chronic inflammation, contributing to 45% of deaths from all causes globally (Wynn, 2004). In the liver, the development of fibrosis plays a crucial role in affecting the quality of life and clinical prognosis of patients (D’Amico et al., 2018b). Liver fibrosis is characterized by the accumulation of extracellular matrix, which destroys the normal physiological structure of the liver (Bataller & Brenner, 2005; Iredale, 2007). The degree of fibrosis progression is closely related to the decline in liver function and is a major risk factor for liver cirrhosis and liver cancer (Roehlen, Crouchet & Baumert, 2020). According to statistics, liver cirrhosis is currently the 11th leading cause of death worldwide and the fourth leading cause of death among adults in Central Europe (Asrani et al., 2019; D’Amico et al., 2018a; Marcellin & Kutala, 2018).

Scientific discoveries over the past decade have transformed our understanding of the mechanisms of liver fibrosis, showing that fibrosis can be reversible, particularly when the disease-causing factors are eliminated or controlled (Albanis & Friedman, 2001; Bataller & Brenner, 2005; Jarcuska et al., 2010; Soyer, Ceballos & Aldrete, 1976). In light of this, predicting the risk of liver fibrosis plays a crucial role in diagnosis and prognosis, as well as guiding treatment decisions during the monitoring of patients with chronic liver disease (Chen et al., 2020; Sang et al., 2021). There are now several non-invasive tools, such as liver elastography, magnetic resonance elastography, and serum biomarkers, to assess and predict liver fibrosis (Li et al., 2016; Moosavy et al., 2023). However, there are still not many tools that function as screening methods for high-risk individuals to optimize cost-effectiveness, especially in middle- and low-income countries (Jiang et al., 2022). A practical and inexpensive model that utilizes only simple, routinely available variables could therefore serve as a cost-effective basis for determining which individuals warrant more detailed diagnostic evaluations.

In Vietnam, chronic liver disease is becoming increasingly prevalent. It is closely associated with common risk factors such as alcohol consumption and chronic hepatitis B and C virus infections, particularly among men (Gish et al., 2012; Nguyen et al., 2022; Nguyen, Law & Dore, 2008). However, there is no nationwide approach or systematic community-based screening for at-risk individuals (Gish et al., 2012). This gap in preventive strategies contributes to the rising risk of progression to severe stages such as liver fibrosis/cirrhosis, liver cancer (Gish et al., 2012; Luu et al., 2022; Thong & Quynh, 2021), as well as related cardiovascular and kidney complications (Dang et al., 2024; Nguyen et al., 2025). In this context, we have selected male adults - a high-risk group with many complex factors influencing disease progression, such as high alcohol consumption and high rates of hepatitis B and C, along with genetic and environmental factors to investigate the current state of liver fibrosis.

Building on this context, the present study focused on developing a simple and easy-to-apply risk prediction model for liver fibrosis based on routinely available clinical variables. The research hypothesis was that simple clinical variables such as age, alcohol abuse, hepatitis B or C infection, hepatic steatosis, and family history of cirrhosis can be used with reasonable accuracy to predict the presence of liver fibrosis in this population.

## Materials & Methods

### Study design and population

This study was a cross-sectional, double-center study conducted in 2021 in Can Tho Central General Hospital, Can Tho City, and Bac Lieu Center City, Bac Lieu Province, Vietnam. These major provincial hospitals are located in the Mekong Delta region in southwest Vietnam.

**Figure 1:**
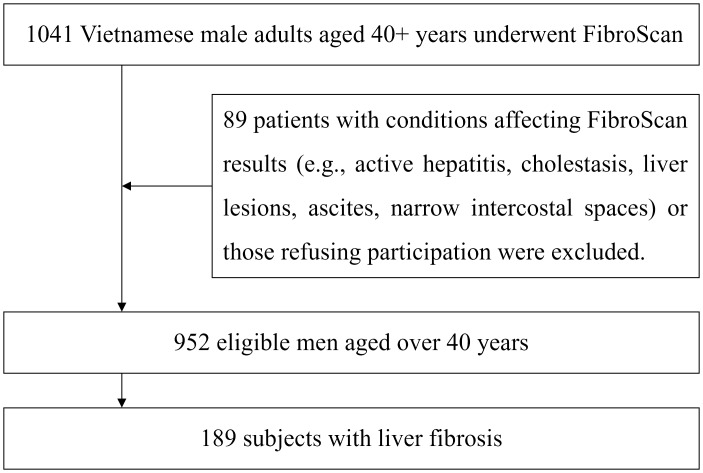
Flow diagram of study participants.

The present study included male participants over 40 years old who had been indicated for FibroScan to assess the degree of liver fibrosis at the recruitment. These patients did not have conditions that could affect the FibroScan results, such as active hepatitis, cholestasis, localized lesions in the measurement area (*e.g.*, liver tumors, liver cysts, liver abscesses), or specific issues that could interfere with the results, such as a large amount of abdominal fluid or excessively narrow intercostal spaces or did not agree to participate in the study. The flow of patient recruitment and exclusion is presented in Fig. 1.

The study’s procedure and protocol were approved by the research and ethics committee of Can Tho University of Medicine and Pharmacy and 121 Military Hospital, Can Tho City, Vietnam (approval number: 546/PCT-HÐÐÐ). The study was conducted according to the ethical principles of the Declaration of Helsinki, and all participants gave written informed consent.

### Data collection

A standardized questionnaire was used to collect demographic and clinical data. The anthropometric parameters included age, weight and height. Clinical variables included a history of alcohol abuse, a history of hepatitis B and C virus infection, hepatitis B vaccination, fatty liver disease, and a family history of cirrhosis. Body weight was measured using an electronic balance in indoor clothing without shoes. Height was determined without shoes on a portable stadiometer with a mandible plane parallel to the floor. The body mass index (BMI) was derived as the weight in kilograms divided by the square of the height in meters. Based on the calculated BMI, participants were categorized as overweight (BMI 23.0–24.9 kg/m^2^), obese I (BMI 25.0–29.9 kg/m^2^), and obese II (BMI ≥ 30.0 kg/m^2^) according to the WHO classification criteria for Asia-Pacific populations (Girdhar et al., 2016). Alcohol abuse is defined as alcohol consumption exceeding 60 g/day (Aberg et al., 2023; Jophlin et al., 2024), sustained until the diagnosis of liver disease (this corresponds to >60 g of alcohol, approximately equivalent to six 330 mL bottles of beer 4%, six 100 mL glasses of wine 13.5%, or six 40 mL servings of spirits 30%). Chronic hepatitis B virus (HBV) infection is defined by persistent HBsAg positivity for more than 6 months and HBV DNA levels exceeding 2,000 IU/mL (Terrault et al., 2018). Chronic hepatitis C virus (HCV) infection is defined by persistent anti-HCV positivity for more than 6 months and detectable HCV RNA in the serum (Yau, Marquez-Azalgara & Yoshida, 2015). Hepatitis B vaccination status is determined based on the patient’s vaccination records. Liver steatosis was diagnosed using conventional ultrasound imaging, including both alcoholic fatty liver disease (AFLD) and nonalcoholic fatty liver disease (NAFLD) (Rinella et al., 2023). Family history of cirrhosis is determined based on medical records or self-reported information from study participants.

The liver fibrosis assessment process was performed using the FibroScan 502 machine with the M probe from Echosen, France. According to the manufacturer’s recommendations, the measurement result is the average of 10 measurements and is displayed in kPa (kilo Pascal). The liver elasticity values range from 2.5 to 75 kPa. The results are immediate and do not depend on the interpreting physician. Depending on the degree of liver fibrosis, this index is classified from F0 to F4, corresponding to the Metavir scoring system. Specifically, F0 (no fibrosis/no scarring), F1, mild to moderate fibrosis (portal tract fibrosis without septa formation/minimal scarring); F2, significant fibrosis (portal tract fibrosis with infrequent/rare septa formation); F3, severe/advanced fibrosis (numerous septa, but no cirrhosis); F4, cirrhosis (cirrhosis/advanced scarring) (Bedossa & Poynard, 1996).

The prevalence of liver fibrosis was determined by the number of cases classified as F1 or higher per 100 people. Initially, the dataset was randomly split into a training set (70%) and a testing set (30%) to allow for model development and preliminary performance assessment. There were no missing data in the study variables, thus no imputation procedures were required. First, the Bayesian model averaging (BMA) method based on binary logistic regression was applied to identify the most optimal models for the eight predictor variables. From the three models with the highest posterior probabilities, we recorded the variable combinations of Model I (the model with the highest posterior probability), which also demonstrated the lowest Bayesian Information Criterion (BIC) value, further supporting its parsimony and goodness of fit. Then, we applied the forward stepwise method to sequentially add variables from the remaining two models to the model with the highest posterior probability until we achieved the maximum number of statistically significant variables. This approach takes advantage of both BMA (the ability to evaluate the best combinations of variables from various possibilities) and forward stepwise (the ability to explore additional potentially useful variables), while retaining the strength of the BMA method in minimizing the risk of overlooking important variables, as can happen with the stepwise method due to its highly linear approach to evaluating variables one at a time (Raftery, Madigan & Hoeting, 1997). The included variables in the forward-stepwise analysis were age, BMI, history of alcohol use, hepatitis B and C virus infection, hepatic steatosis, hepatitis B vaccination, and a family history of cirrhosis.

For internal validation, a bootstrap resampling procedure with 2,000 iterations was performed. The discriminative ability of the final model was evaluated using the receiver operating characteristic (ROC) curve analysis and the corresponding area under the curve (AUC) with 95% confidence intervals (95% CI) estimated by bootstrap resampling. Model calibration was further examined by calibration plots, and overall accuracy was quantified by the Brier score, both estimated using the same bootstrap resampling approach (Nah et al., 2021). When evidence of miscalibration was detected, model updating was explored by applying a restricted cubic spline recalibration procedure to the original linear predictor, thereby allowing for flexible correction of non-linear deviations in the calibration curve without altering the original set of predictors (Croxford, 2016). The analyses were conducted using the R Statistical Environment (version 4.5.0; R Foundation for Statistical Computing, Vienna, Austria). Data preprocessing and management were performed using the dplyr and tidyr packages to ensure transparent and reproducible handling of raw data. Variable selection was first approached with the BMA package through Bayesian Model Averaging, which allowed for weighted evaluation of candidate predictors. Subsequently, multivariable logistic regression modeling was carried out using stepwise selection (implemented in the MASS package) to derive the final parsimonious predictive equation. For each independent predictor retained in the final model, odds ratios (OR) with corresponding 95% CI were estimated to facilitate clinical interpretability. Model performance was comprehensively assessed. Discrimination was quantified by AUC, with 95% CI estimated *via* bootstrap resampling (pROC and boot packages). Calibration was evaluated using the rms package, including graphical assessment with calibration plots and numerical indices (*e.g.*, Brier score). Calibration curves were visualized with ggplot2, and for recalibrated models, spline-smoothed calibration lines were additionally displayed to illustrate improvements in model fit. All statistical analyses were performed in a clean R session to guarantee full reproducibility (Harrell, 2015; R Core Team, 2020).

## Results

Among the 952 participants in the study, the majority were aged 46–55, accounting for 85.2%. Notably, the BMI category of overweight and obesity comprised the majority, with about half of the participants classified as obese (Grade 1) and about one-third as overweight. Regarding associated risk factors, more than 50% consumed alcohol, and more than one-third had fatty liver disease. The rate of hepatitis B infection was nearly six times higher than that of hepatitis C, which may be partly due to the very low vaccination rate for hepatitis B (only 4.1%) (Table 1).

**Figure 2:**
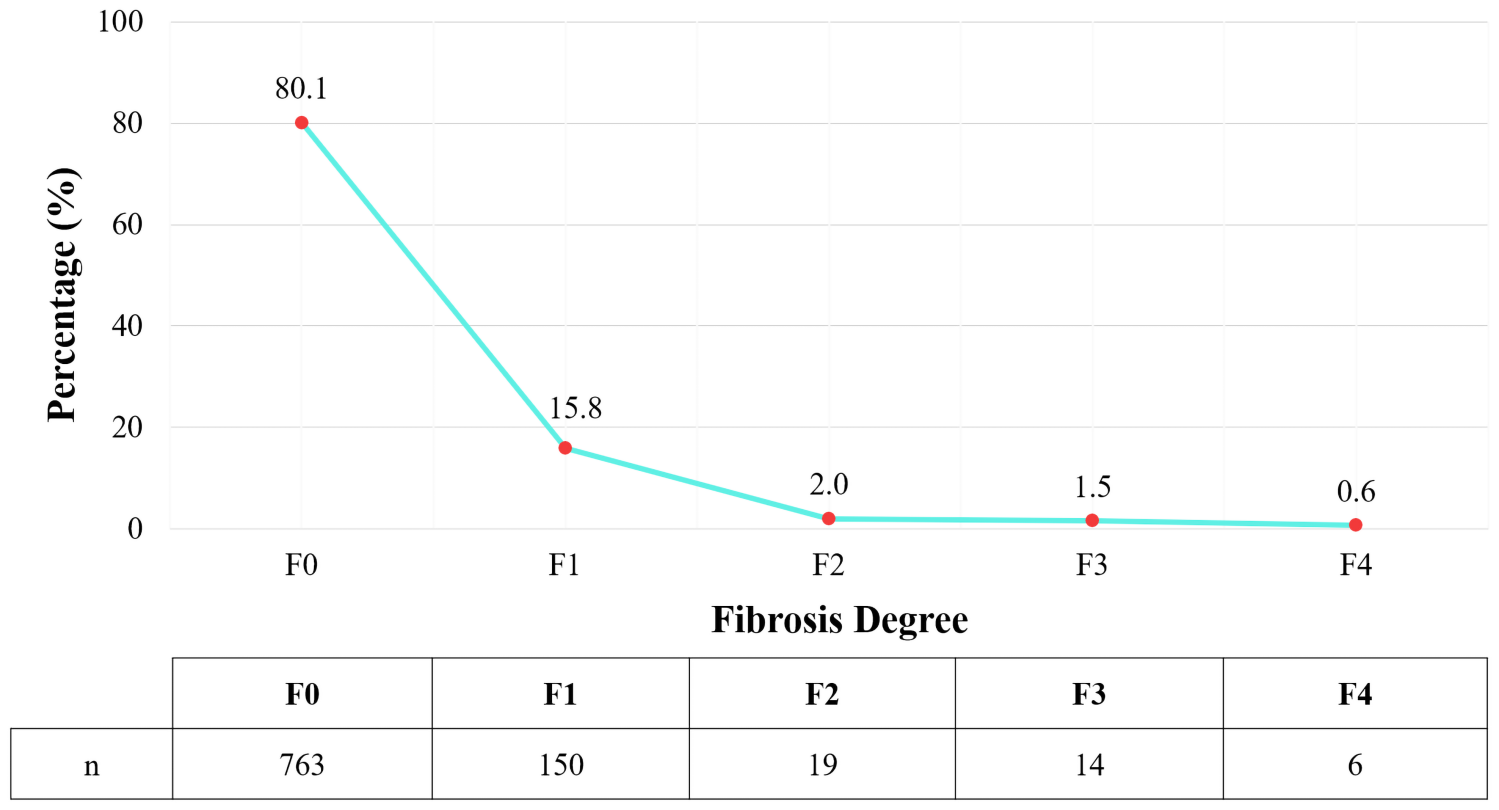
Distribution of liver fibrosis degree according to Metavir classification.

The study recorded 189 subjects with liver fibrosis, corresponding to a rate of 19.9%. Among them, most cases were of mild liver fibrosis (F1) (shown in Fig. 2).

Multivariable analysis revealed advancing age (OR = 1.6; 95% CI [1.02–2.51]), alcohol abuse (OR = 4.44; 95% CI [2.65–7.42], *p* < 0.001), hepatitis B virus infection (OR = 6.76; 95% CI [3.14–14.54]), hepatitis C virus infection (OR = 33.04; 95% CI [5.26–207.42]), family history of cirrhosis (OR = 16.14; 95% CI [3.28–79.55]) and hepatic steatosis (OR = 4.02; 95% CI [2.57–6.28]) were independently associated with the risk of prevalent liver fibrosis (Table 2).

**Figure 3:**
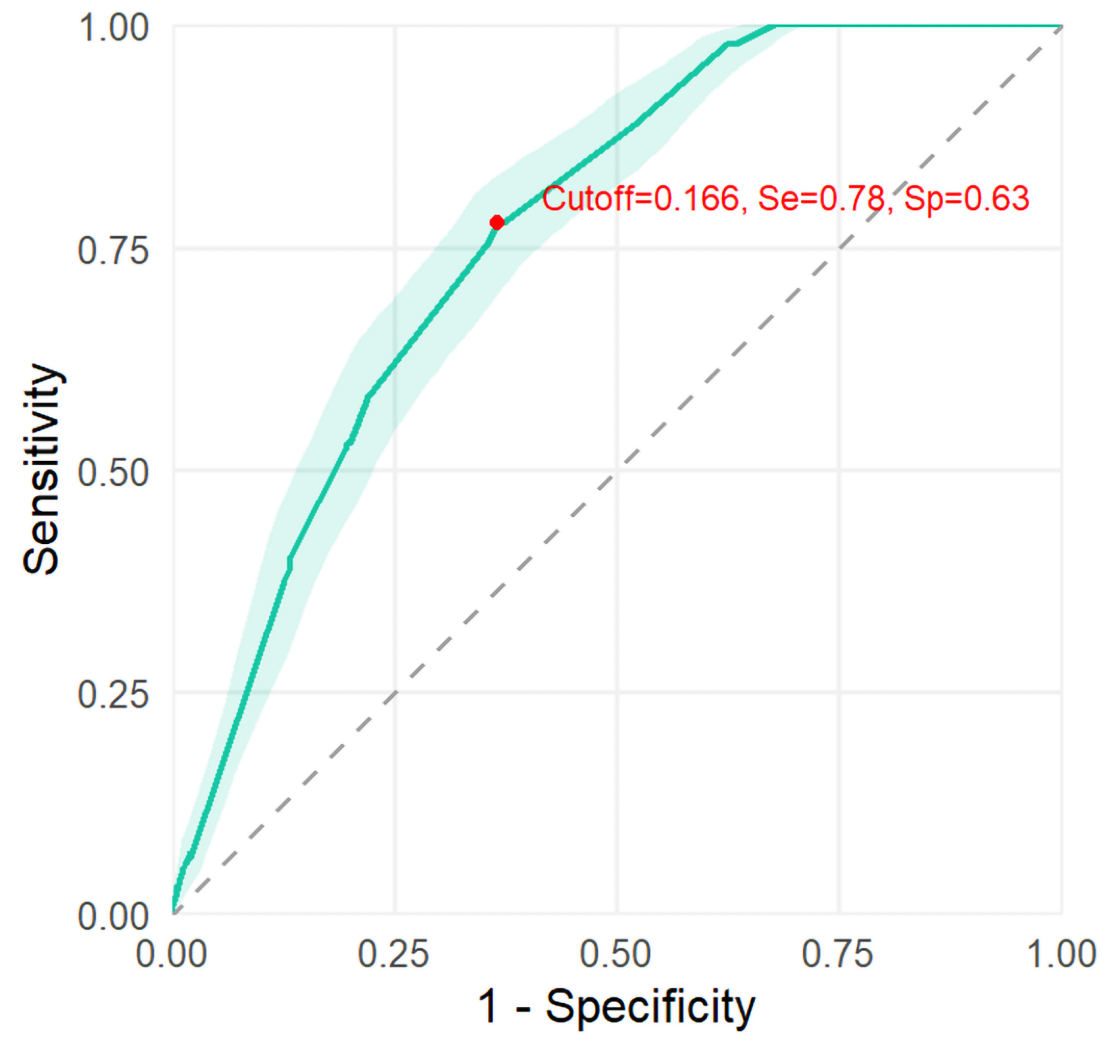
The discrimination of the predictive model.

**Figure 4:**
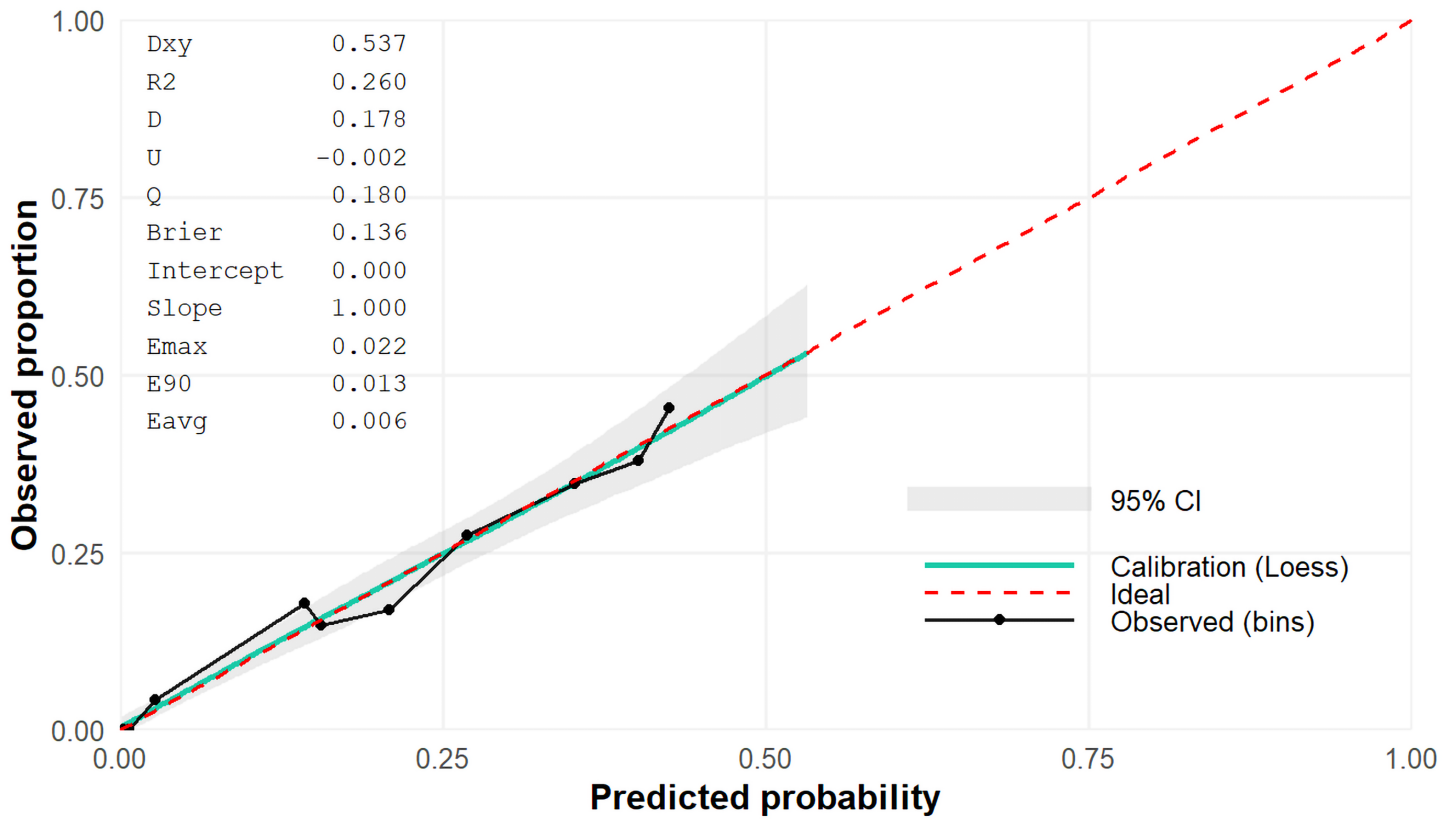
Calibration plot with Brier score of the predictive model.

In order to evaluate the discriminative performance of the predictive model, an AUC value was shown of 0.769 (95% CI [0.734–0.800]) (shown in Fig. 3). Additionally, the calibration curve demonstrated good agreement between predicted and observed probabilities, supporting the reliability of the model’s predictions (Fig. 4).

**Table 1:** Demographic characteristics and risk factors of 952 participants.

**Demographic** ** characteristics**		**Value**
Age (years)	41–45 (n, %)	44 (4.6)
	46–50 (n, %)	310 (32.6)
	51–55 (n, %)	501 (52.6)
	56–60 (n, %)	97 (10.2)
	Mean ± SD (years)	51.4 ± 3.3
BMI (kg/m^2^)	Underweight (n, %)	3 (0.3)
	Healthy weight (n, %)	148 (15.5)
	Overweight (n, %)	279 (29.3)
	Obese class I (n, %)	466 (48.9)
	Obese class 2 (n, %)	56 (5.9)
	Mean ± SD (kg/m^2^)	25.1 ± 2.7
Alcohol abuse	Yes (n, %)	513 (53.9)
Hepatitis B virus infection	Yes (n, %)	66 (6.9)
Hepatitis C virus infection	Yes (n, %)	11 (1.2)
Hepatitis B virus vaccinated	Yes (n, %)	39 (4.1)
Family history of cirrhosis	Yes (n, %)	20 (2.1)
Hepatic steatosis	Yes (n, %)	367 (28.6)

**Table 2:** Multivariate analysis of the risk factors for liver fibrosis and the corresponding equation.

**Factor**		**Unit** ** of** **comparison**	**Coefficients**	**Odds** ** ratio** **(** **95% CI** **)**	***p*** **-value**
A	Age	>50 years old	0.47	1.6 (1.02–2.51)	0.04
AA	Alcohol abuse	Yes	1.48	4.38 (2.66–7.2)	<0.001
B	Hepatitis B virus infection	Yes	1.68	5.38 (2.49–11.63)	<0.001
C	Hepatitis C virus infection	Yes	3.10	22.21 (4.55–108.48)	<0.001
F	Family history of cirrhosis	Yes	2.12	8.35 (2.14–32.64)	0.002
H	Hepatic steatosis	Yes	1.19	3.28 (2.12–5.06)	<0.001
Log$ \left( \frac{p}{1-p} \right) $= −3.46 + 0.47 (A) + 1.48*(AA) + 1.68 (B) + 3.10*(C) + 2.12*(F) + 1.19*(H)					

## Discussion

Liver fibrosis often progresses silently and is typically diagnosed at advanced stages, such as cirrhosis or severe complications. Early diagnosis and treatment can halt or even reverse this progression, highlighting the importance of developing predictive models for liver fibrosis to reduce the associated disease burden. In our study of 952 male participants, approximately two out of 10 individuals were diagnosed with liver fibrosis, most of which were mild cases. Significant risk factors associated with an increased likelihood of liver fibrosis included age, alcohol abuse, infection with hepatitis B or C viruses, family history of cirrhosis, and hepatic steatosis (*p* < 0.05). Based on these factors, a simple predictive model for liver fibrosis in men was developed with reasonably good accuracy.

To date, very few studies have investigated the prevalence of liver fibrosis in the general population. Our study’s prevalence of liver fibrosis aligns with previous global reports, ranging from 0.7−5.7% (Harris et al., 2017). Community-based studies in Europe and the United States reported prevalence rates of approximately 18% (Caballería et al., 2018; Kim et al., 2021), with non-alcoholic fatty liver disease being the leading cause. Similarly, population-based studies across Asia documented a wide variation, from 2.8% in China to nearly 10% in South Korea and Japan, and up to 18% in Romania (Man et al., 2023; Nagaoki et al., 2022; Nah et al., 2021; Trifan et al., 2023). Notably, the prevalence of significant fibrosis or cirrhosis in our study (4.1% and 0.6%, respectively) was lower than that reported in France, Italy, and Hong Kong (Petta et al., 2018; Roulot et al., 2011; Wong et al., 2012). The etiology of fibrosis also differs across populations: non-alcoholic fatty liver disease predominates in many regions, whereas alcohol-related and viral hepatitis remain important causes in others (Altamirano-Barrera, Barranco-Fragoso & Méndez-Sánchez, 2017; Bruha, Dvorak & Petrtyl, 2012). In Vietnam, where HBV infection affects an estimated 8.4 million individuals (Dunford et al., 2012), our study found HBV prevalence nearly six times higher than HCV. Furthermore, over half of participants reported regular alcohol consumption, and nearly 85% were overweight or obese, with fatty liver disease accounting for one-third of cases. These findings highlight the interplay of viral hepatitis, lifestyle factors, and metabolic risk in the burden of liver fibrosis, underscoring the importance of early diagnosis and intervention.

The current study identified six major independent risk factors associated with liver fibrosis in men, including age over 50, alcohol abuse, hepatitis B and C infections, family history of cirrhosis, and hepatic steatosis. Aging is considered a leading factor contributing to liver diseases in general and cirrhosis in particular (Georgieva, Xenodochidis & Krasteva, 2023). Our findings indicate that age > 50 significantly increases the risk of cirrhosis in males. A study by Nah et al. in a Korean population demonstrated a significant association between age and liver fibrosis (OR = 1.34 *p* < 0.001). Moreover, older age was also linked to more severe disease progression (*p* < 0.001) (Nah et al., 2021). Similarly, in a survey of 31,327 individuals in Japan, Kariyama et al. (2024) reported that liver fibrosis severity, assessed using the FIB-3 index, increased with age. Specifically, among individuals aged ≤ 50 years, more than 90% had FIB-3 < 1.5, indicating minimal fibrosis. However, in those aged > 50 years, the proportion of individuals with FIB-3 ≥ 1.5 increased progressively, reaching approximately 30% in the 71–75-year age group. Notably, severe cirrhosis (FIB-3 ≥ 2.5) was observed in more than 10% of individuals aged ≥ 65 years (Kariyama et al., 2024). These findings are fully consistent with, and further supported by, data from multiple previous studies (Koehler et al., 2016; Pitisuttithum et al., 2020). Fibrosis results from a complex interplay between genetic predisposition, aging-related factors, and molecular mechanisms (Georgieva, Xenodochidis & Krasteva, 2023). As aging progresses, hepatocyte quantity and volume decline, liver arterial walls thicken, blood and bile flow deteriorate, and cellular and molecular mechanisms regulating liver regeneration are impaired (Wahid et al., 2024). These changes contribute to progressive liver damage and ultimately lead to fibrosis. Thus, our study not only reconfirms but also reinforces the central role of aging as a universal and independent determinant of liver fibrosis across populations. In our study, alcohol abuse is also a significant risk factor for liver fibrosis, which is further corroborated by existing data. Research in Japan has shown that frequent alcohol consumption—defined as daily drinking or consuming ≥ 60 g of ethanol multiple times per week—is associated with higher FIB-3 index levels compared to non-drinkers (Kariyama et al., 2024). Similarly, Askgaard et al. (2015) reported that men who consumed alcohol every day had an odds ratio for liver fibrosis of 3.65 compared to those who drank less frequently. A systematic review by Roerecke et al. (2019) reported that men with heavy alcohol consumption (≥ 5 drinks/day) had a 4- to 7-fold increased risk of developing cirrhosis compared to non-drinkers. Furthermore, in the Framingham study, Rice, Naimi & Long (2023) demonstrated that even moderate-risk alcohol consumption was significantly associated with liver fibrosis (OR = 1.49). Notably, even after excluding individuals with alcohol abuse, the association with fibrosis remained significant (OR = 1.16). Conversely, a study conducted in South Korea did not identify any significant association between alcohol consumption and the risk of liver fibrosis. This discrepancy may be attributed to the study’s lower threshold for defining excessive alcohol consumption, which was set at ≥ 14 and ≥ 7 standard drinks per week for men and women, respectively (Nah et al., 2021). In reality, while over 90% of heavy drinkers develop alcoholic fatty liver disease, only about 30% progress to severe alcoholic hepatitis and fibrosis/cirrhosis (Ceni, Mello & Galli, 2014). This difference reflects the influence of factors like sex, genetics, and co-factors such as viral hepatitis and obesity on alcohol-induced liver injury. In susceptible individuals, chronic alcohol consumption triggers pathological responses, including acetaldehyde toxicity, oxidative stress, and inflammation through the gut-liver axis (Ceni, Mello & Galli, 2014; Gao & Bataller, 2011; Szabo & Bala, 2010). These processes cause hepatocellular damage, activate hepatic stellate cells, and promote collagen production, ultimately leading to liver fibrosis. Our findings therefore strengthen the evidence that alcohol abuse, especially at high doses, remains one of the most preventable yet potent drivers of fibrosis progression. The study also found that chronic hepatitis B virus and hepatitis C virus infections were significantly associated with liver fibrosis. In the large Rotterdam cohort study, Koehler et al. (2016) reported that chronic HBV infection (defined by hepatitis B surface antigen positivity) or chronic HCV infection (anti-HCV positivity) increased the risk of liver fibrosis by 5.38 times. Similarly, in the national survey on fatty liver and fibrosis in China, Man et al. (2023) observed that the prevalence of severe fibrosis and cirrhosis was at least twice as high in HBV-infected individuals compared to healthy controls. From another research in France, Pol et al. showed that the proportion of patients with advanced fibrosis was significantly higher in those with HCV mono-infection (52.0%) compared to those with HBV mono-infection (32.0%). Further analysis revealed that HCV infection significantly increased the risk of severe fibrosis in HBV-infected patients (OR = 3.84). However, co-infection did not exacerbate fibrosis more than HCV mono-infection, indicating that HCV is the primary driver of fibrosis progression in co-infected individuals (Pol et al., 2017). HBV and HCV induce liver fibrosis primarily through chronic inflammatory responses and persistent hepatocellular injury. Specifically, HBV and HCV cause direct liver cell damage through viral replication while simultaneously stimulating cellular immune responses and releasing proinflammatory cytokines. These factors activate hepatic stellate cells, increasing collagen production and progressive fibrosis (Boltjes et al., 2014; Higashi, Friedman & Hoshida, 2017; Wu, Xu & Lu, 2014). Taken together, our study confirms viral hepatitis as a central etiological factor of fibrosis in Asian populations, which is particularly relevant in Vietnam where HBV prevalence remains high. A family history of cirrhosis was another factor associated with liver fibrosis. In a study on familial heritability and the risk of liver fibrosis, Caussy et al. (2017) found that the prevalence of advanced fibrosis was significantly higher among first-degree relatives of individuals diagnosed with NAFLD-related cirrhosis compared to controls (17.9% *vs.* 1.4%, *p* = 0.0032). Genetic predisposition to liver fibrosis is largely influenced by gene variants involved in lipid metabolism, inflammation, and immune response. 64–68 Variants in genes such as PNPLA3 and TM6SF2 have been identified as increasing the risk of fibrosis by affecting lipid metabolism and fat accumulation in the liver (Caussy et al., 2017; Parola & Pinzani, 2024; Ramos-Lopez et al., 2015). Additionally, genetic factors may heighten liver susceptibility to injury and fibrosis upon exposure to environmental factors such as alcohol consumption, viral hepatitis, or obesity (Ramos-Lopez et al., 2015). This highlights the potential role of gene–environment interactions in shaping fibrosis risk and underscores the importance of family-based screening in high-prevalence countries. Finally, hepatic steatosis was found to be closely associated with liver fibrosis in this study. Koehler et al. (2016) reported an increased fibrosis risk in individuals with fatty liver (OR = 1.99), which was higher in those with diabetes (OR = 5.20). A large Chinese study showed that fatty liver disease is an independent predictor of all stages of fibrosis, with affected individuals having about three times the prevalence of fibrosis compared to those without it (Man et al., 2023). The underlying mechanism involves oxidative stress and chronic inflammation due to hepatic steatosis, leading to hepatocyte damage, hepatic stellate cell activation, collagen synthesis, and fibrotic tissue buildup in the liver (Basaranoglu, Basaranoglu & Sentürk, 2013). Our findings therefore support the growing recognition that steatosis, particularly when coupled with metabolic risk factors, acts as a pivotal early driver of the fibrotic cascade. Overall, the six risk factors identified in the present study are among the most well-established contributors to liver fibrosis and disease progression.

Based on identified risk factors, the study developed a predictive model for liver fibrosis in men using easily obtainable and well-recognized variables, making it a practical tool for identifying high-risk individuals. Bayesian Model Averaging and stepwise regression were used to construct the optimized model. Initially, BMA identified the best variable combinations, while stepwise regression incorporated additional predictors from the remaining models into the highest-probability BMA model, maximizing statistical significance. This approach effectively combined the strengths of both methods, with BMA evaluating variable combinations and stepwise regression allowing flexible screening of individual variables. The final model showed good discriminatory performance, achieving an AUC of 0.769 (95% CI [0.734–0.800]) based on bootstrap resampling. To our knowledge, our model is comparable to other previously reported fibrosis prediction models. For instance, the fibrosis prognosis model developed by El-Kamary et al. (2013) for chronic hepatitis C patients included variables such as hyaluronic acid, TGF-β1, α2-macroglobulin, Matrix Metalloproteinase (MMP)-2, apolipoprotein-A1, urea, MMP-1, alpha-fetoprotein, haptoglobin, red blood cell count, hemoglobin, and TIMP-1. This model achieved high accuracy, with an AUC of 94.9% (95% CI [91.3–97.4]) and 89.8% (95% CI [82.9–94.6]) for the training and validation sets, respectively. Similarly, Sang et al. (2021) developed a liver fibrosis prediction model for NAFLD patients from China, Malaysia, and India using nine parameters, including age, BMI, fasting blood glucose, diabetes status, alanine aminotransferase (ALT), γ-glutamyl transferase, triglycerides, and the aspartate transaminase (AST)-to-platelet ratio. The AUC of this model was 0.82 for distinguishing early and advanced fibrosis (sensitivity = 0.69, specificity = 0.80) in the discovery cohort, and its diagnostic performance remained robust in two independent validation cohorts (AUC = 0.89 and 0.71) (Sang et al., 2021). While these models demonstrated better predictive performance than ours, they have limitations, such as using smaller patient populations or including numerous variables that require complex lab tests, which may not be easily accessible in routine clinical practice, making them harder to apply in initial disease management. In addition, Chen et al. (2020) developed a noninvasive model based on laminin, procollagen III N-terminal peptide, and platelet count, which achieved an AUC of 0.765 for predicting significant fibrosis in Chinese patients with chronic hepatitis B. Notably, despite relying on specialized biochemical markers, the discriminative ability of their model was still lower than that of ours. In contrast, Boursier et al. (2017) developed the easy liver fibrosis test (eLIFT) for chronic liver disease patients, which includes age, sex, gamma-glutamyl transferase, AST, platelet count, and prothrombin time to detect advanced fibrosis. The eLIFT model showed a good AUC for detecting severe fibrosis (0.781 ± 0.013), with a sensitivity of 78.0% at a cutoff value of ≥ 8 (Boursier et al., 2017). Likewise, Lindvig et al. (2025) created the LiverPRO model using UK Biobank data, incorporating age and three to nine variables from a panel of nine simple blood tests (AST, alkaline phosphatase, gamma-glutamyl transferase, albumin, sodium, bilirubin). LiverPRO demonstrated strong performance in detecting clinically significant fibrosis with an AUC of 0.86 for development and 0.80 for validation cohorts. This result was comparable to the Enhanced Liver Fibrosis test (AUC = 0.78) and LiverRisk score (AUC = 0.81) while outperforming the Fibrosis-4 index (AUC = 0.69) and NAFLD fibrosis score (AUC = 0.74) (Lindvig et al., 2025). The latter two models are well suited for early fibrosis prediction, sharing similarities with our model regarding the acceptable number of variables, ease of clinical data collection, and good predictive accuracy. In fact, in Vietnam and many other developing countries, primary healthcare facilities often lack advanced laboratory equipment, making models that rely on simple clinical and demographic parameters especially valuable for early identification and preventive strategies. Therefore, this model may serve as a practical starting point for clinical and community-based applications, especially in preventive medicine where accessibility and feasibility are critical. Importantly, by enabling preliminary risk stratification with minimal resources, our model could also serve as a cost-effective basis for deciding whether more detailed diagnostic evaluations are warranted. In addition to discrimination assessed by the AUC, our analysis also incorporated calibration, thereby offering a more comprehensive appraisal of model performance. The predicted probabilities closely matched the observed outcomes, with only minor deviations from the ideal reference line. This indicates that the model not only distinguishes effectively between risk groups but also generates clinically reliable probability estimates. Although other reference models have demonstrated similarly acceptable calibration (Chen et al., 2020), their reliance on a larger panel of specialized biomarkers may reduce their feasibility for widespread use, especially in primary care or resource-limited settings. These considerations do not necessarily imply superiority of our model, since differences in patient populations and the lack of direct head-to-head comparison preclude definitive conclusions. Nonetheless, our model exhibited acceptable discrimination and robust calibration. Furthermore, the internal validity of these findings was reinforced through bootstrap resampling, underscoring the model’s stability. Looking ahead, however, external validation across independent cohorts remains essential to establish generalizability and confirm its applicability in diverse clinical contexts.

These findings reflect the substantial burden of liver fibrosis among Vietnamese men, with key risk factors including age over 50, alcohol abuse, HBV and HCV virus infection, family history of cirrhosis, and hepatic steatosis. These data underscore the importance of identifying and managing these risk factors, which remain significant public health challenges in Vietnam. There is a pressing need to implement effective screening, prevention, and treatment programs to reduce the current disease burden and prevent the long-term consequences of chronic liver disease on public health. Finally, we have successfully developed a predictive model for liver fibrosis in men that allows individualized risk assessment for liver fibrosis based on key clinical characteristics. The model is designed to be simple, relying on easily obtainable risk factors, making it particularly suitable in resource-limited settings where access to advanced diagnostic technologies is restricted. This approach enables the early identification of high-risk individuals who may benefit from more in-depth diagnostic testing.

This study has several limitations that should be acknowledged. The current sample size may not fully represent Vietnamese men or the general population. A liver biopsy was not performed for direct histological assessment in any of the patients, resulting in the absence of a gold standard to validate the model’s predictive outcomes. Most importantly, this predictive model has not yet been validated in independent populations, leaving its real-world performance in the general population uncertain and requiring further evaluation in future studies.

## Conclusions

Our population-based study showed that the prevalence of liver fibrosis was approximately 20% among Vietnamese male participants, with most cases being mild. Independent risk factors for liver fibrosis identified were age > 50, alcohol abuse, hepatitis B or C infections, family history of cirrhosis, and hepatic steatosis. A simple predictive model developed based on these risk factors demonstrated fairly good predictive performance, highlighting its potential clinical utility in identifying male individuals at high risk for liver fibrosis who would benefit from timely screening and intervention. However, the study has certain limitations, including the lack of external validation, the cross-sectional design that does not allow causal inference, and the representativeness of the sample restricted to middle-aged and older Vietnamese males, which may limit the generalizability of the findings. Future research should consider validating the model in independent populations with different demographic and geographic characteristics, including women and younger individuals, and explicitly examining possible interactions among predictors to further enhance predictive power, interpretability, and clinical applicability.

##  Supplemental Information

10.7717/peerj.20435/supp-1 Supplemental Information 1 Raw data Patient identifiers, the variables used in the model—age, alcohol abuse, hepatitis B, hepatitis C, family history of cirrhosis, hepatic steatosis—and the liver fibrosis stage classified according to the METAVIR system

10.7717/peerj.20435/supp-2 Supplemental Information 2 STROBE checklist

## Additional Information and Declarations

### Data Availability

The following information was supplied regarding data availability: The raw data is available in the Supplementary File.
